# Development and Validation of the Cognitive Behavior Questionnaire in Nonspecific Chronic Low Back Pain (CBQ-NSCLBP)

**DOI:** 10.7759/cureus.41853

**Published:** 2023-07-13

**Authors:** Seema Saini, Paresh Golwala, Devashish Tiwari

**Affiliations:** 1 Physiotherapy, Sumandeep Vidyapeeth, Vadodara, IND; 2 Physiotherapy, Dr. D.Y. Patil Vidyapeeth, Pune, IND; 3 Orthopaedics, Sumandeep Vidyapeeth, Vadodara, IND; 4 Physiotherapy, Massachusetts General Hospital Institute of Health Professions, Boston, USA

**Keywords:** questionnaire, self-report measure, behavioral factors, cognitive factors, chronic pain, validity, reliability, psychometric properties, validation, cognitive behavior questionnaire development

## Abstract

Background

It’s common to experience chronic low back pain (CLBP), having serious psychological as well as physical implications. The experience of CLBP and its management depends greatly on cognitive and behavioral aspects. However, there is a requirement for a valid and reliable instrument that assesses the characteristics associated with this condition.

Purpose

The Cognitive Behavior Questionnaire in Nonspecific Chronic Low Back Pain (CBQ-NSCLBP), a questionnaire for measuring cognitive behavior was created and validated for this study.

Methodology

To validate the instrument, the consensus technique with two rounds of the modified Delphi process was used. With a varied background, 13 experts in the field were specifically chosen. Utilizing the literature review, an outline of the questionnaire was created. Each of the suggested things was evaluated by the experts using a five-point Likert scale. The items completed the descriptive analysis using the criteria for validation and elimination.

Results

The research findings showed that the CBQ-NSCLBP had good psychometric characteristics. The content Validity Ratio (CVR) (Critical) for a panel size (N) of 13 is 0.54 & for CBQ-NSCLBP is 0.70, which was regarded as indicative of strong content validity. Test-retest reliability showed that intra-class correlation coefficient (ICC) values for all the items were greater than 0.9 which indicates excellent reliability. Spearman’s correlation test between CBQ-NSCLBP and the Survey of Pain Questionnaire-Brief (SOPA-B) has shown that all the items are valid since the correlation value is higher than the cutoff value (0.139). Exploratory Factor Analysis was performed and three factors were retained based on eigenvalue greater than one.

Conclusion

In order to evaluate cognitive behavior in Nonspecific Chronic Low Back Pain (NSCLBP), we created this tool. This will make it easy to understand the level of cognition in NSCLBP patients.

## Introduction

At some point in life, almost 80% of people will face low back pain (LBP). LBP affects 48% of Indians, with females having the highest prevalence [1]. Research suggests that 8 to 15% of individuals with LBP have a pathoanatomical diagnosis, leaving the rest with Nonspecific Chronic Low Back Pain (NSCLBP) [2,3]. When lower back pain continues after three months of symptomatic onset, it is considered as chronic leading to increased medical costs, lost productivity, and a decline in quality of life [4].

A number of Randomized Control Trials have investigated that cognitive behavioural intervention can successfully treat NSCLBP [5-7]. The conventional biomedical view of LBP has been critically questioned over the last decade. Multifaceted treatment is needed in patients with NSCLBP [5-7]. The evidence suggests that NSCLBP is a disorder in which impairment is more directly related to the behavioural and cognitive aspects [8-10]. NSCLBP is a multifaceted problem that includes one's physical condition, psychological/cognitive, and social participation [11,12]. A vicious cycle of pain arises from NSCLBP because it causes maladaptive cognitive behaviours such as negative views, fear, and catastrophizing [11,13].

Previous research suggests a strong association between psychological distress, catastrophizing, fear-avoidance belief, and NSCLBP [4,14]. Analysis and interpretation of these associated factors are very important when we are treating NSCLBP [15-20]. While many assessments concentrate on evaluating symptoms or overall functioning, a deeper understanding of the underlying cognitive causes causing discomfort or dysfunction is required. It is necessary to conduct a thorough assessment of cognitive distortions and maladaptive thought patterns because these aspects may be overlooked by other approaches [9,10]. Therefore, this study aimed to develop and validate the CBQ-NSCLBP (Cognitive Behavior Questionnaire in NSCLBP) using a modified Delphi iterative survey approach.

The findings of the study's content validity were presented at the 2023 Combined Sections Meeting (CSM) in San Diego, California, on February 23, 2023, and an abstract of the presentation was published in the conference proceedings of the Journal of Orthopaedic & Sports Physical Therapy.

## Materials and methods

The study was conducted at the Outpatient Department (OPD) of Dr. D.Y. Patil College of Physiotherapy in Pune. Convenience sampling was used to recruit participants for the study. Under the number Trial REF/2020/12/039472, this study was registered with Clinical Trials Registry-India (CTRI). Sumandeep Vidyapeeth Institutional Ethics (SVIEC) has granted this study ethical approval and assigned it the SVIEC/ON/Phys/PhD/20027 reference number. Dr. D. Y. Patil College of Physiotherapy also granted institutional sub-ethical clearance for this study, with reference number DYPCPT/ISEC/24/2020.

One hundred and seventy-four patients of NSCLBP were selected based on the inclusion criteria of persistent pain for at least three months that was aggravated by changes in posture, movement, or physical activity. All the participants between the age of 18-60 years were included [16,17]. Participants were excluded if they received a medical procedure for pain relief (such as a facet or sacroiliac joint injection, myofascial trigger point injection, or denervation procedure) within the last three months, had prior spinal surgeries, had indications of neurological damage (i.e. reduced reflexes or motor deficits), and definable spinal pathology (e.g. malignancy, fracture, infection, spinal stenosis, spondylolisthesis, inflammatory joint or bone disease) and abnormal bowel and bladder function [18,19].

Questionnaire development

To investigate how cognitive factors affect non-specific chronic low back pain, a literature review was done. The keywords "cognitive behavior outcome," "chronic pain," "chronic low back pain," and "evaluation" were entered into the Medline, Pubmed, and CINAHL databases. Thirty-five items were initially compiled from the literature review.

Modified Delphi process

The responses received from the multidisciplinary experts (physiotherapists, orthopedic surgeons, general practitioners, and clinical psychologists) with a minimum of five years of clinical and academic experience were gathered using the modified Delphi technique. It was determined to have at least 13 responses in the first round of the modified Delphi procedure from the aforementioned group of specialists. The same 13 experts were invited to participate in the second round of the modified Delphi Process based on the replies provided in the first round. All 13 experts gave their consent to be recognized as experts in the modified Delphi procedure.

In the first round of the modified Delphi method, participants were asked to score each item's acceptability for inclusion in the evaluation of cognitive variables in NSCLBP patients on a 5-point Likert scale (where 1 meant Not at all Important and 5 meant Extremely Important). Following each question, respondents were given a spot where they were asked to add free text comments, reiterate points, or offer other ideas that they felt would be useful to include if applicable. Demographic data, educational background, current position, years of experience, and respondents' self-rated level of process competence were also requested utilizing an online form.

The round 1 survey of modified Delphi was conducted using an online Google Form and 35 items questionnaire was sent to experts. The primary investigator sent invitations to participate in the modified Delphi process to about 18 experts through email, and 13 of those experts have responded indicating their interest. In order to improve participation in the survey, this was followed up by up to two phone calls. Written informed consent was obtained from the experts who participated in the modified Delphi process. After incorporating the suggestions from experts and content validity scores 16 items questionnaire was constructed; the mean/median score of the entire panel, as well as the individual score of each respondent were sent to the experts during the second round of the modified Delphi Process. Respondents either maintained or reconsidered and allotted a new score if required. The final scores were presented to the expert panel. Sixteen items that are rated 4 or more by 80% of respondents were considered for final acceptance. A final questionnaire of 16 items was then tested on 174 patients diagnosed with NSCLBP.

Outcome measures

CBQ-NSCLBP (Cognitive Behavior Questionnaire in Non-Specific Chronic Low Back Pain)- 5-point Likert scale was used to evaluate the 16-item scale (0 being not at all true and 4 being all the time true). With an overall CVI (Content Validity Index) of 0.86, which indicated excellent content validity, and a Cronbach's alpha score of 0.76, which showed adequate internal consistency, the initial analysis indicated potential psychometric characteristics. This scale was tested on 174 patients for test-retest reliability (Table 1).

**Table 1:** CBQ-NSCLBP after round 2 of modified Delphi CBQ-NSCLBP: Cognitive Behavior Questionnaire in Nonspecific Chronic Low Back Pain; LBP: Low Back Pain

S. No.	Questions	Not at all (score 0)	To a slight degree (score 1)	To a moderate degree (score 2)	To a great degree (score 3)
1	Do you get tired easily due to LBP?				
2	Do you get restless and unable to hold still in sitting, standing or lying more than 30 minutes due to LBP?				
3	Do you still love things that you used to do before LBP?				
4	Does LBP disturb the clarity of your thoughts?				
5	Does episode of LBP increase feeling of your guilt of not effectively being able to participate in work most of the time?				
6	Does an episode of LBP make you feel like crying at times?				
7	Do you feel more depressed than usual because of LBP?				
8	Does LBP affect your sleep?				
9	Do you feel that your LBP will never end?				
10	Are you afraid that the LBP may worsen with time?				
11	Do you feel that you can’t keep LBP out of your mind?				
12	Do you feel your LBP gets worse with physical activity?				
13	Do you think that your LBP was caused by the work?				
14	Do you think you can ever go back to work with your LBP?				
15	Do you think that your work may make your LBP severe?				
16	Do you think until your LBP is treated, you cannot do your usual work?				

## Results

Participants

The study's participants had an average age of 42.77 (8.71) years and 174 patients with NSCLBP completed the outcome measure (Table 2).

**Table 2:** Demographic data

Age	Frequency	Percentage
20-30	15	8.62%
31-40	49	28.16%
41-50	70	40.23%
51-60	38	21.84%
61-70	2	1.15%
Total	174	100.00%

Content validity

For a panel size (N) of 13, the Critical Validity Ratio (CVR) was 0.54, and for the Cognitive Behavior Assessment Questionnaire (CBAQ), it was 0.75. These numbers were thought to be good content validity indicators. The CBAQ's overall Content Validity Index (CVI), equal to 0.88, demonstrated excellent content validity [21].

Factor analysis

Kaiser-Meyer-Olkin measure of sample adequacy value was 0.609 (values > 0.5 are regarded acceptable), and the Bartlett's Test of Sphericity value of 239.179 was significant (P.001), indicating that the items were suitable for factor analysis [22].

**Figure 1:**
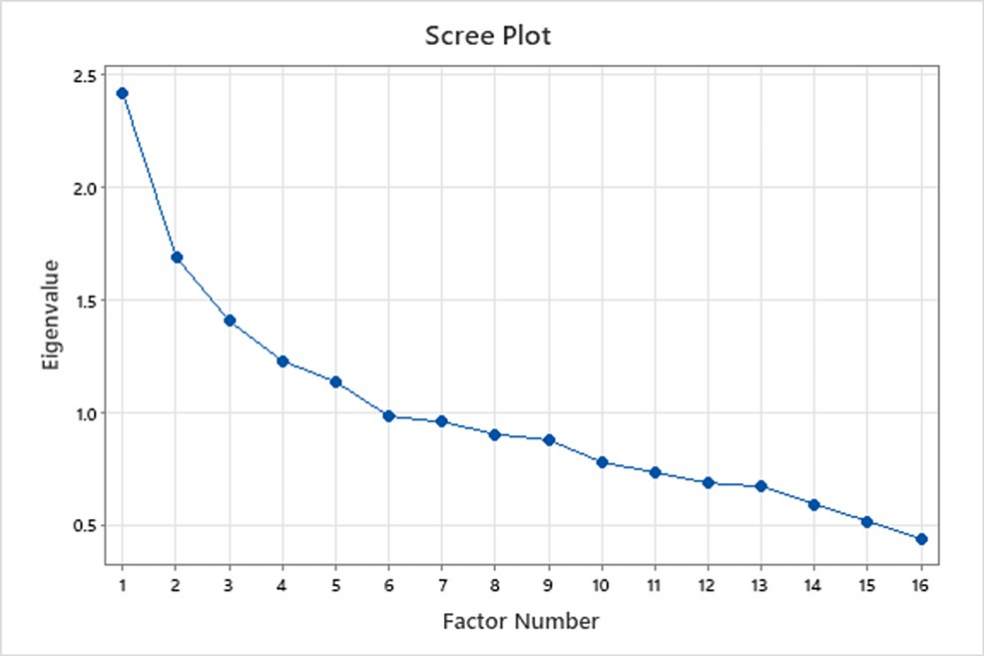
Scree plot showing Eigenvalues for items

The component number and eigenvalues are plotted on a scree graph. The scree plot makes it clear that the graph's line starts to become flat after the third component. This suggests that the subsequent components are making up a smaller percentage of the overall variation. In this situation since these three components are the ones that contribute the most variance (Figure 1).

To identify the underlying patterns and relationships among the CBQ-NSCLBP components, an exploratory factor analysis (EFA) with oblique rotation was carried out. The Oblique rotation (Promax) is used for obtaining the rotated solution. Oblique rotation such as Promax produces the factor pattern and the factor structure matrix. In this investigation, the three factors with the highest eigenvalues (greater than 1) were retrieved. The relationship between the factors and the original items is revealed by the items' loading pattern. According to the loading pattern, items V11, V16, V5, V8, V7, V4, and V3 showed better loading on factor 1, items V2, V6, V1, and V9 showed better loading on factor 2, and items V15, V10, V12, V14, and V13 showed better loading on factor 3. (Table 3).

**Table 3:** Structure matrix with loading values using exploratory factor analysis Extraction Method: Principal Component Analysis; Rotation Method: Promax with Kaiser Normalization.

	Factors		
	1	2	3
(V11) Do you feel that you can’t keep LBP out of your mind?	0.637	0.003	0.031
(V16) Do you think until your LBP is treated, you cannot do your usual work?	0.545	-0.162	0.084
(V5) Does episode of LBP increase feeling of your guilt of not effectively being able to participate in work most of the time?	0.544	0.278	0.202
(V8) Does LBP affect your sleep?	0.490	0.420	-0.220
(V7) Do you feel more depressed than usual because of LBP?	0.453	0.266	0.006
(V4) Does LBP disturb the clarity of your thoughts?	0.425	0.155	0.082
(V3) Do you still love things that you used to do before LBP?	0.344	0.017	-0.207
(V2) Do you get restless and unable to hold still in sitting, standing or lying more than 30 minutes due to LBP?	0.087	0.639	0.007
(V6) Does an episode of LBP make you feel like crying at times?	0.366	0.554	0.026
(V1) Do you get tired easily due to LBP?	-0.080	0.553	0.311
(V9) Do you feel that your LBP will never end?	0.230	0.464	0.248
(V15) Do you think that your work may make your LBP severe?	0.091	0.075	0.668
(V10) Are you afraid that the LBP may worsen with time?	0.049	-0.062	0.533
(V12) Do you feel that your LBP gets worse with physical activity?	-0.112	0.210	0.531
(V11) Do you feel that you can’t keep LBP out of your mind?	0.283	-0.398	0.465
(V16 Do you think until your LBP is treated, you cannot do your usual work?	0.051	0.170	0.436

Test-retest reliability

Using IBM SPSS Statistics 26.0 (IBM Corp., Armonk, Survey), the intra-class correlation coefficient (ICC) estimates and 95% confidence intervals were calculated. Based on the mean rating, absolute agreement, and a 2-way mixed effect model, the ICC estimates were derived. All of the items' ICC values were greater than 0.9 (0.993-0.998), which is an indication of excellent reliability. The overall ICC value was 0.996, which is significant at the 1% significance level between the total score on days 1 and 2 [23].

Internal consistency

Each subscale and the entire CBQ-NSCLBP were examined for internal consistency using Cronbach's alpha. Cronbach's alpha scores of 0.74 to 0.76 were regarded as being suggestive of adequate internal consistency [24].

## Discussion

The creation and validation of the Cognitive Behavior Questionnaire in NSCLBP are done in this study. The questionnaire demonstrated acceptable construct validity, good reliability, acceptable internal consistency, and good content validity. The rigor of the investigation has been further increased by using statistical analyses and expert opinions. There are significant similarities and consistency between the study's findings and the literature already available on chronic non-specific low back pain (CNLBP). Modified Delphi method which is commonly utilized in the development of questionnaires to acquire expert opinions and ensure that the items properly represent the measured construct. The inclusion of expert consensus in the selection and modification of items aligns with the best practices in questionnaire development [23-25].

Factor structure

The study used EFA to identify the underlying components of the questionnaire. We have identified three factors classified as psychological stress, fear avoidance belief, and catastrophizing. This finding corresponds with existing literature that has identified several factors contributing to cognitive-behavioral aspects of CNLBP [26,27].

Internal consistency

Through the use of Cronbach's alpha and ICC values, the questionnaire's reliability was assessed. The questionnaires showed acceptable internal consistency. This result is consistent with earlier research that assessed the validity of comparable questionnaires in CNLBP populations [28].

Test-retest reliability

The high ICC values (>0.9) also suggest excellent test-retest reliability, indicating that the questionnaire provides consistent results over time. Comparable reliability values have been reported in previous studies evaluating the reliability of pain-related questionnaires [29]. The developed questionnaire demonstrated content validity, reliability, and construct validity, consistent with previous research findings [29]. These consistencies reinforce the relevance of cognitive-behavioral factors in understanding and managing CNLBP and highlight the value of using validated questionnaires to assess these factors in clinical and research settings. Further studies comparing the developed questionnaire with established measures of cognitive-behavioral factors in CNLBP would provide additional insights into its discriminative validity and potential advantages in clinical practice.

The fact that the study was restricted to a specific geographic area may limit the application of the results to other cultural settings or populations. Consequently, it is vital to acknowledge that the psychometric properties and applicability of the CBQ-NSCLBP may differ across various settings. Although the modified Delphi method is widely used to establish content validity, it is dependent on expert opinions, which may introduce subjectivity.

Clinical implications

The developed questionnaire can serve as a useful resource for researchers and practitioners in evaluating cognitive behavior aspects in those who experience NSCLBP. It has the potential to facilitate understanding of the psychological factors associated with pain perception. Moreover, the factors identified through EFA can guide future research in exploring specific dimensions of cognitive behavior in this population.

Implications for future research

Future research can improve the validity, reliability, and responsiveness of the created CBQ-NSCLBP by undertaking thorough analyses employing Rasch analysis and item response approaches. These investigations will support better clinical judgment and patient care.

## Conclusions

As CLBP has a strong relationship with both mechanical and psychological aspects. In order to evaluate cognitive behavior in NSCLBP, we created this tool. In particular, psychological distress, catastrophizing, and fear avoidance belief in patients with NSCLBP can be examined using the CBAQ, which has shown excellent validity.
